# Hybrid GMR Sensor Detecting 950 pT/sqrt(Hz) at 1 Hz and Room Temperature

**DOI:** 10.3390/s18030790

**Published:** 2018-03-06

**Authors:** André Guedes, Rita Macedo, Gerardo Jaramillo, Susana Cardoso, Paulo P. Freitas, David A. Horsley

**Affiliations:** 1 Picosense Inc., Berkeley, CA 94704, USA; andre.guedes@picosense.com (A.G.); rita.macedo@picosense.com (R.M.); dahorsley@ucdavis.edu (D.A.H.); 2 INESC Microsystems and Nanotechnologies and IN-Institute of Nanoscience and Nanotechnology, 1000-029 Lisbon, Portugal; scardoso@inesc-mn.pt (S.C.); Paulo.Freitas@inl.int (P.P.F.); 3 Instituto Superior Tecnico, Universidade de Lisboa, Av. Rovisco Pais, 1000 Lisbon, Portugal; 4 Berkeley Sensor and Actuator Center, University of California, Davis, CA 95616, USA

**Keywords:** GMR, MEMS, picotesla, low frequency, heart rate

## Abstract

Advances in the magnetic sensing technology have been driven by the increasing demand for the capability of measuring ultrasensitive magnetic fields. Among other emerging applications, the detection of magnetic fields in the picotesla range is crucial for biomedical applications. In this work Picosense reports a millimeter-scale, low-power hybrid magnetoresistive-piezoelectric magnetometer with subnanotesla sensitivity at low frequency. Through an innovative noise-cancelation mechanism, the 1/f noise in the MR sensors is surpassed by the mechanical modulation of the external magnetic fields in the high frequency regime. A modulation efficiency of 13% was obtained enabling a final device’s sensitivity of ~950 pT/Hz^1/2^ at 1 Hz. This hybrid device proved to be capable of measuring biomagnetic signals generated in the heart in an unshielded environment. This result paves the way for the development of a portable, contactless, low-cost and low-power magnetocardiography device.

## 1. Introduction

Over the past decade, the development of ultrasensitive magnetic field sensors at low frequencies has attracted intense research [1,2,3,4], with emphasis for biomedical applications such as magnetocardiography (MCG) and magnetoencephalography (MEG). The most well-known highly sensitivity magnetometer has been the Superconducting Quantum Interference Device (SQUIDs), with noise levels of 5 fT/Hz^1/2^ [5]. SQUIDs require cryogenic refrigeration to operate, which significantly increases their size, power consumption and cost. Other alternative magnetic sensor technology that have witnessed substantial developments are the atomic magnetometers. Early in 2000, a spin-exchange relaxation-free (SERF) atomic magnetometer (AM), with non-cryogenic operation and magnetic field sensitivity of 0.54 fT/Hz^1/2^ (in the range of 28–45 Hz) was demonstrated [6]. Since then, further advancements enabling the demonstration of SERF AM for imaging of brain activities [7] and the development of compact optically pumped atomic magnetometers (OPMs) prototypes aiming at commercialization have been reported [8,9]. 

MR sensors have been at the forefront of research in the development of ultra-sensitive magnetic sensors [10,11]. MR sensors are based on thin-film technology, enabling high scalability and they also can be a great alternative to reduce the overall system complexity due to their capability of being fully integrated (in particular with CMOS). MR technology has evolved to provide greater magnetic field sensitivity, more compact size, higher tolerance to environmental conditions, and better electrical performance combined with lower price and high functionality. However, the use of MR technology for ultra-low magnetic field detection suffers from 1/f noise that causes a reduction of their limit of detection by three orders of magnitude in the low-frequency regime (<10 Hz), critical for biomedical applications. Different strategies have been pursued to minimize this type of noise in MR sensors [12,13,14,15]. Edelstein et al. [16] introduced the concept of microelectromechanical (MEMS) system flux concentrators with the potential to solve the problem of 1/f noise. Using this approach, the magnetic field at low frequency is modulated to higher frequency with vibrating MEMS resonator integrated with magnetic flux concentrators, whose role is to focus and amplify the external field in the sensor area [17]. Several results on 1/f noise reduction with these hybrid MR-MEMS devices have been reported. Using a pair of magnetic flux concentrators driven by electrostatic combs Edelstein et al. [18] was able to perform a thousand-fold 1/f noise reduction in a spin-valve GMR sensor. Guedes et al. also developed hybrid sensors integrating electrostatic cantilevers driving flux guides with GMR sensor, with a reported modulation efficiency of 0.11% [19]. Later on, by integrating two MEMS piezoelectric cantilevers that incorporated magnetic flux concentrators (MFC) on top with a GMR sensor, a modulation efficiency of 1.6% was obtained [20]. Here, we move a step further by demonstrating an improved efficiency of 13% with a hybrid GMR—piezoelectric cantilevers MEMS, leading to a device with a final sensitivity of 950 pT/Hz^1/2^.

**Figure 1:**
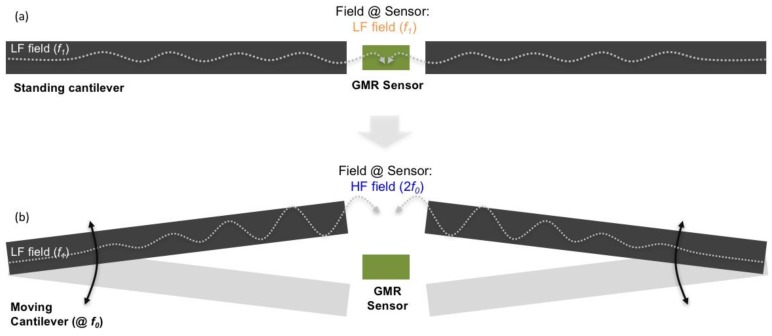
Simplified schematics of the device (side view) (**a**) in a standby position or (**b**) when cantilevers are moving. The cantilevers mechanical movement induces an AC magnetic field at the GMR sensor region. The field is detected at high frequency, where the sensor is not limited by 1/f noise.

The proposed device combines giant magnetoresistance (GMR) sensors, magnetic flux concentrators (MFC), and MEMS cantilevers. The MEMS piezoelectric cantilevers incorporating MFC modulate the low frequency magnetic signals above 100 kHz, where the 1/f noise in the GMR sensor can be neglected [16,17,18,19,20]. The result of this modulation is a shift in the operation frequency of the hybrid GMR-MEMS device, leading to an improvement of the detection limit from approximately 1 nT/Hz^1/2^ (low frequency—1/f noise limited) to 1 pT/Hz^1/2^ (high frequency—thermal noise limited). Figure 1 shows schematics of the device (side view). The external low-frequency field at frequency *f*_1_ (or DC field) flows through the flux concentrators on the MEMS cantilevers. The cantilever’s mechanical oscillation at the cantilever’s resonant frequency *f*_0_ acts as a carrier frequency, inducing an oscillatory (AC) magnetic field at frequencies 2*f*_0_ (for an external DC field) or 2*f*_0_ ± *f*_1_ (for an external low-frequency *f*_1_ field), that is detected by the GMR sensor [20]. 

GMR sensors with a spin valve (SV) structure [21,22] typically show a magnetic field detection limit in the range of few hundred nT/Hz^1/2^ to nT/Hz^1/2^, at the thermal noise regime, increasing 2–3 orders of magnitude at low frequency. Details on noise sources in MR sensors can be found in [23]. The sensor performance can be characterized through the field modulation efficiency—*α*, that depends on total displacement of the cantilevers and it is defined as the ratio between the field modulated to high frequency (*B_f_*_0_) and the original low frequency/static magnetic field (*B_f_*_1_):

(1) $$\alpha =\frac{B_{f_{0}}}{B_{f_{1}}}$$

The noise expression of the hybrid MR-MEMS device, *S_B_^SV+MEMS^*, can be generalized as a function of the GMR sensor thermal noise [13].

(2) $$S_{B}^{SV+MEMS}=\frac{1}{S\alpha }\sqrt{4k_{B}TR}=S_{B}^{SV+MEMS}=\frac{S_{B}^{SV}}{\alpha }$$

Note that, the GMR sensitivity *S* already comprises the gain obtain with the incorporation of the magnetic flux concentrators (MFC). 

## 2. Materials and Methods

The micro-fabrication of these devices started on a 150 mm diameter 650 µm thick silicon-on-insulation (SOI) wafer comprising 1 µm buried oxide (SiO_2_) and 2 µm Si. On top of it the piezoelectric stack was deposited in an Endeavor AT sputtering tool: Mo (150 nm) as the bottom electrode and 002-oriented AlN (700 nm) as the active piezoelectric material. The stress of the AlN layer was controlled during deposition to a level close to zero. Next, a 200 nm PECVD oxide was deposited and polished by CMP to provide a smooth surface (average roughness—4 Å) and enable stable GMR sensor deposition. The GMR sensors with a top magnetically-pinned spin valve (SV) structure were deposited by on beam deposition (IBD) in a Nordiko3000 tool [24] with average GMR ratio of 7% and patterned down to a dimension of 40 × 1.5 µm^2^ with the following structure (in nm): Ta[2]/NiFe[2.5]/CoFe[2.5]/Cu[2.2]/CoFe[2.5]/MnIr[10]/Ta[2]. The MFC’s (0.3 µm thick multilayers based on anti-ferromagnetic (AF) coupled soft CoFeB films [25]: Ta[3]/Ru[3]/[CoFeB[3.8]/Ru[1.8]] × 32/CoFeB[3.8]/Ru[5.0]) were also deposited by IBD and defined by lift-off. The MEMS fabrication started with sputtering Al to define the cantilever top electrode. The Mo bottom electrode via was opened by dry etching the SiO_2_ and AlN layers. An 800 nm thick LPCVD oxide mask was defined to pattern the cantilevers by dry etching the SiO_2_/AlN/Mo/Si layers, stopping at the buried oxide layer. After opening the device pads (GMR contacts and cantilever electrodes) the cantilevers were released by backside DRIE.

## 3. Results

**Figure 2:**
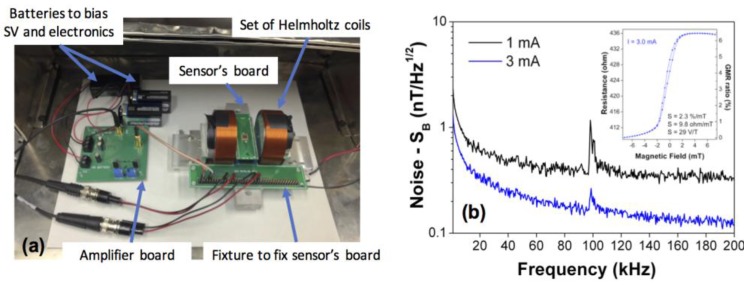
(**a**) Measurement set up used to perform GMR noise measurements; (**b**) Noise spectrum in magnetic units, nT/Hz^1/2^, for different biasing currents in the GMR sensor. The inset shows a GMR sensor transfer curve characterized in the final released device, showing a field sensitivity of 29 V/T when biased at *I* = 3 mA.

Approximately 95% of tested GMR sensors maintained full signal after the MEMS fabrication and final release step. The sensors had an average GMR ratio and resistance of 7% and 500 Ω, respectively, and showed a highly linear magnetic field transfer characteristic. GMR sensor noise measurements were made in a mu-metal shielded box, containing a battery-powered low-noise amplifier (designed using TI OPA211, a fast BiCMOS operational amplifier achieving 1.1 nV/Hz^1/2^ input noise density at 1 Hz), a battery-operated sensor current bias circuit, and a pair of small Helmholtz coils generating a transverse field ranging from −8 mT to 8 mT. Figure 2a shows a picture of the measurement set up used. A SR785 spectrum analyzer was used to acquire the noise power spectral density from DC to 10 kHz, and an Agilent CXA Signal Analyzer was used to acquire the noise from 10 kHz to 200 kHz. Figure 2b shows the GMR sensors’ characterization in terms of noise spectrum on a final micro-fabricated device measured with different biasing currents (*I*). The curves were obtained for the GMR sensor in the absence of an external magnetic field. For a GMR bias current *I* = 3 mA at 200 kHz (near thermal noise), the noise level is of 3.65 nV/Hz^1/2^, which corresponds to a detection limit of 125 pT/Hz^1/2^.

**Figure 3:**
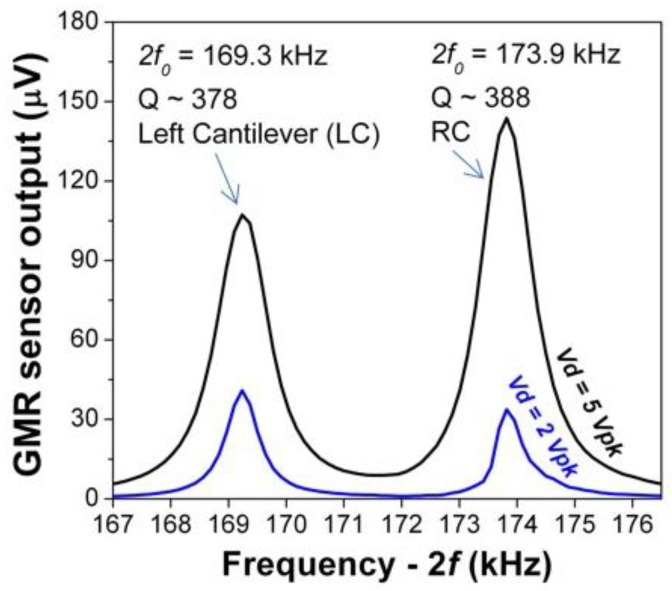
Resonance frequencies of the left (LC) and right (RC) cantilevers detected by the GMR sensor, for two different drive voltages (Vd).

The resonance frequency of the cantilevers was measured by monitoring the GMR output in a presence of a small magnetic field while sweeping the excitation frequency of the MEMS cantilevers. The GMR output is maximized at the resonance of the cantilever by its mechanical Q—factor since the magnetic field modulation depends on the total displacement of the cantilever. Figure 3 shows the resonance frequencies for the left (LC) and right (RC) cantilevers detected by the GMR sensor. The signal was acquired with a lock-in amplifier by sweeping the frequency from *f* = 83 kHz to *f* = 89 kHz and reading the GMR output at 2*f.* It is possible to observe that the resonance frequencies for both cantilevers are slightly offset. This fact was attributed to a dispersion of DRIE micro-fabrication step during the backside release of the cantilevers. In order to achieve the highest field modulation efficiency out of the cantilevers, both LC and RC were driven at the resonance of the RC (2*f*_0_ = 173.9 kHz). To compensate for the low displacement occurring when excited off resonance the LC was driven at very high voltage (V_d_ = 160 V).

**Figure 4:**
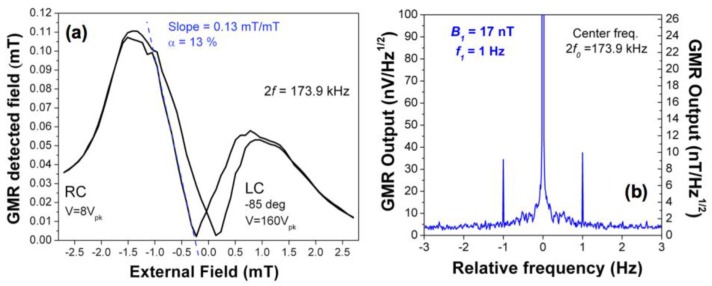
(**a**) Detected field vs applied external field. From the slope of the curve the modulation efficiency (*α*) was calculated—13%; (**b**) Detection of a 17 nT low frequency field (1 Hz). The sidebands’ peak amplitude is approximately ½ of the external applied field amplitude.

In order to measure the modulation efficiency (*α*), the external magnetic field was swept using a small set of Helmholtz coils. Figure 4a shows the resulting hysteresis loop, where *α* can be extracted by the slope of the curve. *α* was found to be 13% for this particular device. This value enables the calculation of the sensitivity of the final hybrid device, using Equation (2), which was found to be 950 pT/Hz^1/2^. The power consumption of the device was largely dominated by the GMR element and calculated to be 3.8 mW. In order to test the performance of the device in the detection of low frequency magnetic fields, the set of Helmholtz coils was used to produce a low amplitude field *B*_1_ at frequency *f*_1_ = 1 Hz and test the modulation and response of the device. The resulting signal is monitored by recording the sidebands 2*f*_0_ ± *f*_1_ with the GMR sensor. The minimum field detectable with our setup was 17 nT, which was successfully measured by our device, and is plotted in Figure 4b. As observed in Figure 4b, the detection peak amplitude is approximately 1/2 the external applied field. 

**Figure 5:**
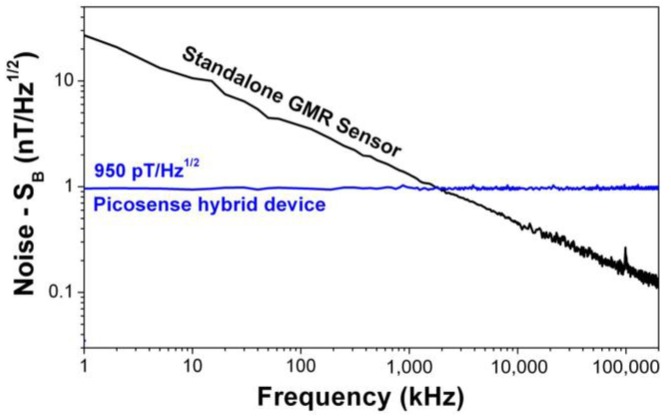
Noise spectrum comparison between a standalone GMR sensor and Picosense’s device. At 1 Hz, Picosense’s device is >10× more sensitive than the GMR sensor.

Figure 5 compares the noise spectrum of a standalone GMR sensor (without MFC elements and MEMS cantilevers) and Picosense’s hybrid sensor. The standalone GMR sensor is clearly affected by the 1/f noise, with the greatest impact observed at low frequencies. The hybrid device developed by Picosense operates at the modulation frequency 2*f*_0_ (hundreds of kHz), therefore the 1/f noise is negligible being the noise of the hybrid device solely limited by modulation efficiency of the cantilevers and the GMR sensor thermal noise at 2*f*_0_. At 1 Hz, Picosense’s hybrid device is >10× more sensitive than a standalone GMR sensor. The GMR sensor alone would not be able to detect the 17 nT magnetic field at 1 Hz, described in Figure 4b. This clearly validates Picosense’s modulation technique and the extremely good low frequency sensitivity of the device. 

This noise level result although representing an improvement when compared with the previous generation [20] still requires further optimization to be able to achieve the theoretical few pT/Hz^1/2^ for the noise level (determined by the thermal noise regime). The final device performance here presented is mainly limited by the mismatched cantilevers displaying slightly different resonance frequencies. This issue implies that while operating the device, one cantilever will be driven at resonance while the second is driven off-resonance, resulting in cantilevers moving with significantly different vertical mechanical amplitude (the cantilever off-resonance with considerable lower amplitude), thus reducing the modulation efficiency of the pair of the cantilevers, one of the major factors reflected in the final device sensitivity. This issue is attributed to microfabrication variations; therefore, efforts on the design and microfabrication aspects are being carried out to minimize the impact of mismatched cantilevers on the final sensitivity of the device.

**Figure 6:**
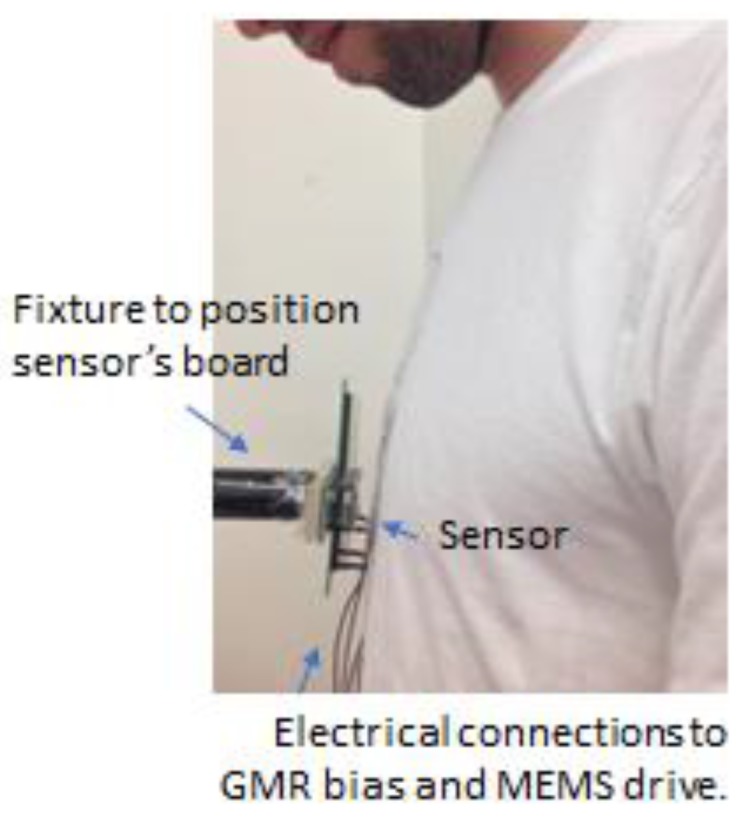
Apparatus used for contactless sensing of the subject heart-rate. The small magnetic sensor die is visible mounted on a chip carrier (sensor’s board). Distance between the sensor and the chest was approximately 1 cm.

**Figure 7:**
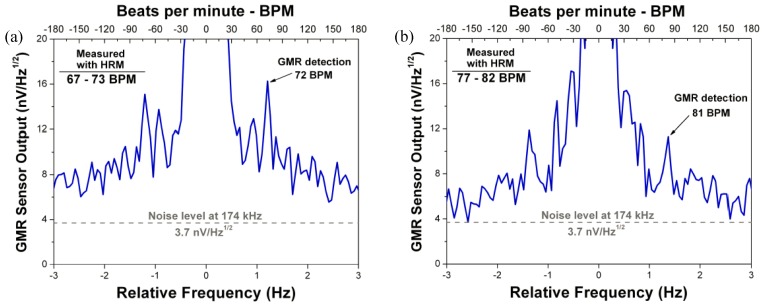
The device developed by Picosense in this project was used to measure heart rate in a subject at rest (**a**) and after a short walk (**b**). The heart-rates were found to be 72 and 81 BPM, respectively, confirmed by a reference heart-rate monitor (HRM).

Furthermore, experiments were performed in order to study the suitability of the device to measure the biomagnetic signals originated in the heart. The measurement was performed with the sensor placed close to the heart, distancing 1 cm to the subject’s chest, in a completely unshielded environment, as Figure 6 depicts. Figure 7a displays the heart-rate of a subject at rest modulated at 174 kHz, the measurement was obtained after 5 min averaging, and repeated several times, to confirm reproducibility. In Figure 7b, the same measurement is repeated after a short walk to increase heart-rate. During the measurement, the heart rate was also monitored with a standard heart-rate monitor (HRM), used as a reference. The heart rate obtained with Picosense’s device is in accordance with the value obtained by the standard HRM, validating our measurement. 

## 4. Conclusions

In this work we have demonstrated the successful fabrication and characterization of a GMR—MEMS hybrid sensor with subnanotesla noise level for low frequency fields ~950 pT/Hz^1/2^ at 1 Hz. It was shown that at 1 Hz, the GMR—MEMS device is >10 times more sensitive than a standalone GMR sensor. These results clearly validate the modulation technique presented here and the extremely good low frequency sensitivity of the device developed. Furthermore, this GMR—MEMS hybrid sensor proved to be a promising candidate for critical biomedical applications, by successfully being capable of measuring the heart-beat from the proximity of a subject chest with no shielding. This result demonstrates the differentiating factor of Picosense’s device in comparison with other devices (e.g., chest straps, portable IR LEDs). Picosense’s device enables heart-rate measurement without any contact and thus avoiding any discomfort and inconvenience to the user. Magnetic fields are not perturbed by typical materials on the optical path of optical sensors such as human sweat. Upon further optimization, with adjustments on the MEMS and device geometry to improve the modulation efficiency and employing higher gain MFCs, this device can achieve sensitivities of the order of few picotesla, potentially enabling the acquisition of the full heart waveform. In conclusion, these results pave the way for the use of this device for portable magnetocardiography (MCG), wherein the high sensitivity, portability and contactless features of this device are attractive and address the limitations of the current technology of the portable electrocardiography (ECG) heart-monitoring market.
